# Pediatric Temporal Lobe Meningioma With Meningioangiomatosis Mimicking Invasive Meningioma

**DOI:** 10.7759/cureus.18819

**Published:** 2021-10-16

**Authors:** Omron Hassan, Hammad Ghanchi, Kennethy DeLos Reyes, Ravi Raghavan, Tanya Minasian

**Affiliations:** 1 Clinical Sciences, Touro University Nevada College of Osteopathic Medicine, Henderson, USA; 2 Neurosurgery, Riverside University Health System Medical Center, Moreno Valley, USA; 3 Neurosurgery, Loma Linda University Medical Center, Loma Linda, USA; 4 Neuropathology, Loma Linda University Medical Center, Loma Linda, USA

**Keywords:** temporal lobe, pediatric, brain tumor, meningioangiomatosis, meningioma

## Abstract

Meningiomas combined with meningioangiomatosis (MA-M) present similarly to more invasive lesions because of their appearance on neuroimaging. These lesions are especially rare in pediatric patients and suggestive imaging can help identify them for differential diagnosis. An 11-year-old male child who presented with diplopia and a headache was found to have an edematous invasive appearing temporal lobe mass on magnetic resonance imaging. Despite the lesion’s appearance, it was completely resected and found to be a benign MA-M upon histopathologic examination. The present case demonstrated a rare meningioma with meningioangiomatosis that appeared to be a higher grade or invasive lesion upon initial imaging in a pediatric patient. A review of the literature was performed on patients who presented similarly. Despite the rarity of this condition in children, neuroimaging should be carefully examined prior to surgical resection of similar masses in preparation for highly vascular tissue, and post-operative course can be better anticipated when MA-M is considered during differential diagnosis.

## Introduction

Interpretation of neuroradiologic findings is critical in the treatment of neurosurgical patients, and benign intracranial masses on magnetic resonance imaging (MRI) may often mimic more invasive lesions due to concomitant presentation. The co-occurrence of meningiomas with meningioangiomatosis (MA) can appear similar to an invasive meningioma on imaging, which presents challenges along the course of treatment. Meningiomas are rarely found in children despite being the most encountered primary neoplasm in the adult population, and few reports in the literature discuss their co-occurrence with MA [1]. MA is a hamartomas mass with potential neoplastic features and is often associated with neurofibromatosis type 2 [2]. MA forms through meningovascular leptomeningeal proliferation but is thought to be benign despite its debated neoplastic features [3].

Occurrences of meningiomas combined with MA (MA-M) are rare and thought to form either through neoplastic transformation of hamartomatous MA or through meningiomas that spread perivascularly along Virchow-Robin spaces [1,4]. MA-M in the pediatric population is usually discovered incidentally or in patients who present with seizures followed by subsequently having intracranial masses found upon imaging [2-8]. MA may also co-occur alongside different forms of tumors other than meningiomas, but when found with meningiomas, they may present radiologically as hypointense lesions with surrounding edema on T2-weighted MRI and diffuse borders suggestive of a higher-grade meningioma.

The authors report a case of an 11-year-old male child who presented with diplopia and a headache. MRI revealed an edematous temporal lobe mass suspected to be an invasive meningioma. But upon surgical resection, the pathologic results found the mass to be a rare World Health Organization (WHO) grade 2 meningioma with accompanying MA. This study aimed to discuss the features of MA-M including its clinical presentation, histopathologic features, and radiologic characteristics through discussion of our present case and a review of the current literature [1-9].

## Case presentation

**Figure 1:**
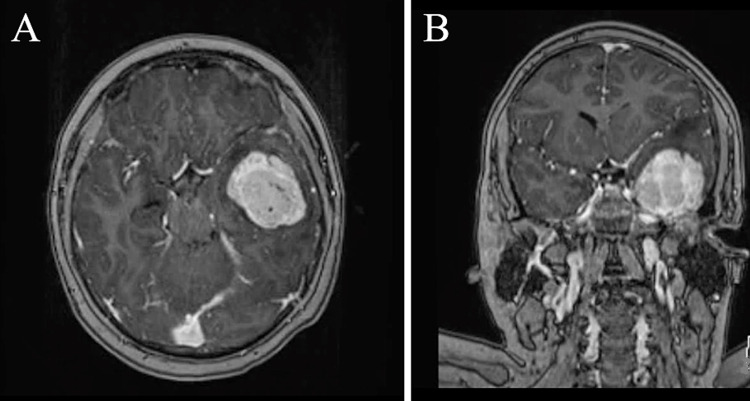
MRI showing an enhancing left temporal tumor T1-weighted axial (A) and coronal (B) MRI showing an enhancing left temporal tumor along the inferior cortical surface with mass effect and significant surrounding vasogenic edema.

An 11-year-old male patient with no significant past medical history or known genetic syndromes presented to the emergency department three days after onset of left frontal headache and associated diplopia. The patient did not have nausea, emesis, paresis, or paresthesia. Physical examination was significant for diminished visual acuity, bilateral papilledema, and anisocoria in the left eye. He also had right pronator drift. He showed no other neurologic deficit. A T1-weighted MRI with contrast showed a heterogeneously enhancing left anterior temporal extraaxial tumor with mass effect and significant surrounding vasogenic edema suggestive of an invasive meningioma, based on its location and appearance (Figure 1). 

**Figure 2:**
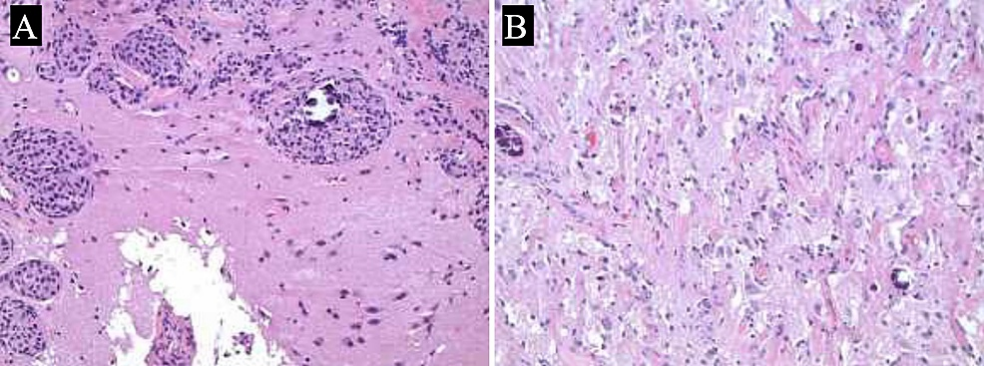
GFAP-stained sections demonstrating meningioma invading cerebral tissue superficially (A) and flanking meningothelial cells entrapping and distorting cortical grey matter (B). GFAP: glial fibrillary acidic protein

Surgical treatment was discussed and determined to be the best option to attenuate symptoms by decompression and obtain diagnosis to optimize long-term outcomes. The patient underwent surgical resection of their tumor through a left pterional craniotomy. Ultrasonic aspiration was used to debulk the tumor centrally followed by circumferential dissection through a plane. The gliotic brain was noted at the periphery of the tumor. No obvious interdigitations of tumor into the brain were grossly noted. Gross total resection was achieved. However, histopathologic examination found meningioma with invading cerebral tissue superficially (Figure 2). 

Histologic sections using glial fibrillary acidic protein (GFAP) immunostaining demonstrated a moderate cellularity meningothelial meningioma with increased mitotic activity. There was also suggestion of early focal necrosis, and high Ki-67 labeling indices were found supporting that this portion of the mass was a WHO grade 2 meningioma with atypical features. In addition, some flanking areas demonstrated distinct disturbances in cerebral parenchyma underlying the meningioma, and a strip of vertically oriented proliferating vessels were seen in vascular columns. This area was also flanked by meningothelial cells that were entrapping and distorting the cortical grey matter between them. This indicated meningioangiomatosis in addition to the mentioned meningioma tissue.

**Figure 3:**
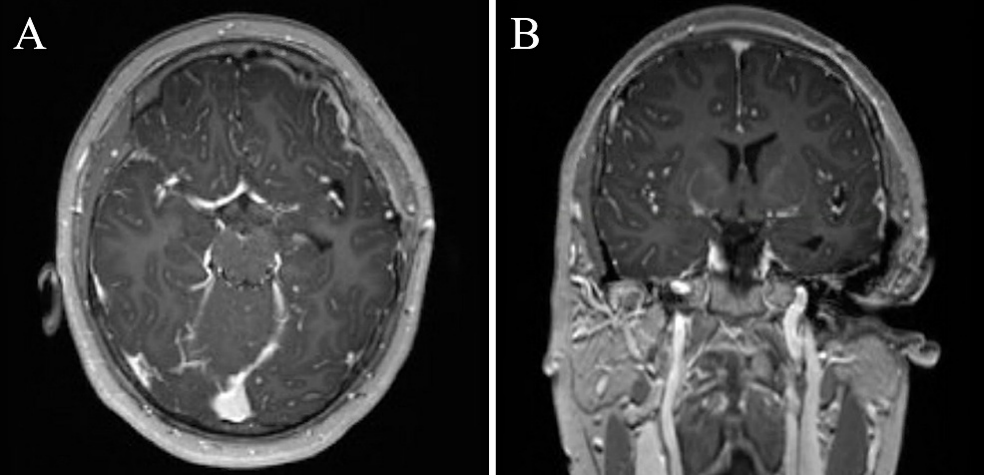
Three-month follow-up T1-weighted MRI axial (A) and coronal (B) views without MA-M recurrence MA-M: meningiomas combined with meningioangiomatosis

There were no postoperative complications and the patient’s presenting symptoms were completely resolved following surgery. The patient’s physical examination improved post-operatively and he was discharged without any sequelae of neurologic deficits. Upon a three-month follow-up, the patient continued to remain asymptomatic and without signs of recurrence on surveillance imaging. There was no plan for adjuvant therapy given no evidence of residual on imaging (Figure 3). 

## Discussion

A review of the English-language literature revealed nine papers published between 1982 and 2020 that reported cases of pediatric MA-M (Table 1) [1-9]. 

**Table 1:** Case reports and series of pediatric MA-M Data from reports of MA-M found during the literature review is summarized. M: male; F: female; MA-M: meningiomas combined with meningioangiomatosis

Publications	Age (years)	N/Sex	Presentation	Main imaging findings	Histopathologic type of meningioma	Complications
Auer et al. 1982 [9]	15	1/M	Headache nausea vomiting	CT showed calcium-containing right frontal and subfrontal mass surrounded by hypodensity	Fibroblastic	Post-operative decerebrate and subsequent death
Blumenthal 1993 [4]	0.8	1/M	Seizure	Focally calcified enhancing lesion in interhemispheric fissure and extending to right frontal lobe on MRI	Transitional	-
Deb et al. 2006 [5]	1.5	1/F	Seizure	Low signal area in left temporal region, isointense on T1 and hyperintense on T2	Transitional	Transient R hemiparesis and infrequent seizures for 8-months
Galloway et al. 2020 [6]	1.5	1/M	Seizure	Cortically based edematous mass within right temporal lobe, mainly hyperintense on T2 and hypointense on T1	Rhabdoid	-
Kim et al. 2002 [7]	3-9	5/M	Seizure	On MRI: round poorly demarcated hypervascular mass, heterogeneous enhancement, well-demarcated ovoid mass with low signal intensity on T2, intracerebral hemorrhage	Transitional fibroblastic meningothelial sclerosing	-
Kim et al. 2009 [2]	3-10	5/M 2/F	Seizure	Well defined calcified masses on brain CT	Fibroblastic transitional meningothelial	Recurrence in one patient
Perry et al. 2005 [8]	0.8-17	5/M 1/F	Seizure	Enhancing or non-enhancing on MRI, appearing similarly to glioma, ganglioglioma, and cortical dysplasia	Transitional fibroblastic meningothelial atypical	-
Sinkre et al. 2001 [1]	8	1/M	Headache nausea vomiting	Ovoid left frontal extra-axial mass that was heterogeneous with vasogenic edema on MRI	Atypical transitional	-
Zhang et al. 2015 [3]	3-13	7/M	Seizure	Variable and nonspecific imaging findings between patients, gyriform alterations were found on MRI, hypointense on T1, hyperintense on T2	Transitional fibroblastic	-

The search found a total of 30 patients, age range 0.8-17 years old, with 26 male and four female. Seizures, headaches, vomiting, and nausea were among the symptoms reported by patients, with seizures being the most common. Imaging findings upon presentation and histopathologic findings of the concomitant meningiomas are summarized in Table 1. Complications included post-operative death [9], transient right hemiparesis [5], seizures [5], and one case of transitional type MA-M recurrence in the temporal region at four-year follow-up [2]. There were no reports of adjuvant chemotherapy or radiation being used. MA-M more commonly occurred in male pediatric patients, and within the general population, male incidence is also more common [6]. Incidence rates of MA-M cannot be determined by current studies, but the few reported cases suggest its rarity and thus more familiarity with presenting neuroimaging findings may allow for optimal surgical planning.

MA may occur alongside meningiomas, but a review by Zhang et al. also found reported cases of MA co-presentation along with arteriovenous malformations, encephaloceles, oligodendrogliomas, meningeal hemangiopericytomas, and focal cortical dysplasia [3]. Transitional and fibroblastic meningiomas most often manifest alongside MA in pediatric patients through our literature review [1-5,7,8]; however, the less encountered forms in this patient population were rhabdoid, sclerosing, meningothelial, and atypical meningiomas [1,2,6-8]. MA appears commonly as meningothelial and fibroblast-like cells that proliferate to infiltrate the leptomeninges with hypercellular areas and sclerosis [6]. In the present case, we found a meningothelial meningioma which is rare in pediatric patients, and the concomitant MA presentation even less commonly encountered. Although these are benign in nature and may lead to excellent outcomes with total resection, caution should be taken when this pathology is suspected based on imaging as heavy bleeding may be encountered intraoperatively because of the hemangiomatous hypervascularity. Preparation for this is especially critical in the pediatric population, where excess blood loss is not tolerated in this young population.

Often a transition zone between the MA and meningioma may be seen on histopathologic examination, and this was similar in structure to the flanking meningothelial cells we found that marked a plane between the meningioma and MA components [3]. Pathologic examination is crucial for adjuvant treatment as it may decide whether radiotherapy or chemotherapy is necessary, thus clear identification of MA-M can affect subsequent patient care following tumor resection [10].

Differentiating presenting intracranial masses on neuroimaging can be based on subtle signs, but in some instances, the appearances between different pathologic subtypes of tumors can mimic one another. Current reports of MA-M suggest that they appear as non-enhancing hypointense masses on T2-weighted MRI [7], but other reports demonstrated hypointense lesions on T1-weighted and hyperintense on T2-weighted MRI [3,5,6]. Poor demarcation along the tumor margins with edema can be found and would suggest hypervascularity akin to that of invasive meningiomas [1,6,7]. MA-M also has been reported to appear with focal calcification on computed tomography (CT) [2,4,9]. MA-M most frequently presents in the frontal area in pediatric patients, but temporal tumors can be rarely encountered as was seen in our patient. Careful consideration of these imaging signs may help narrow down the differential diagnosis to adequately prepare for tumor resection and anticipate heavy bleeding.

## Conclusions

We reported a case of a pediatric patient with MA-M and reviewed related literature. Despite the rarity of MA-M in children, when suggestive imaging signs are encountered, then preparation for highly vascular tissue during resection should be made and post-operative course can be better anticipated. Adequate preparation and careful surgical technique may lead to better clinical outcomes in these patients and avoid potential intraoperative complications.
